# Effects of Multivitamin and Multimineral Supplementation on Blood Pressure: A Meta-Analysis of 12 Randomized Controlled Trials

**DOI:** 10.3390/nu10081018

**Published:** 2018-08-03

**Authors:** Kelei Li, Chunxiao Liu, Xiaotong Kuang, Qingxue Deng, Feng Zhao, Duo Li

**Affiliations:** 1 Institute of Nutrition and Health, Qingdao University, Qingdao 266021, China; likelei@qdu.edu.cn (K.L.); 2017021629@qdu.edu.cn (C.L.); 2017021621@qdu.edu.cn (X.K.); 2017021646@qdu.edu.cn (Q.D.); fzhao@qdu.edu.cn (F.Z.); 2 Department of Food Science and Nutrition, Zhejiang University, Hangzhou 310058, China

**Keywords:** multivitamins, multiminerals, hypertension, blood pressure, meta-analysis

## Abstract

Previous studies have not drawn a consistent conclusion about effect of multivitamin and multimineral supplementation (MVMS) on blood pressure. A comprehensive search of PubMed, Embase and Cochrane Library (up to May 2018) and references of relevant articles was undertaken. The present meta-analysis included 12 randomized controlled trials (RCTs), of which eight RCTs in 2011 subjects evaluated the effect of MVMS on blood pressure and four RCTs in 21,196 subjects evaluated the effect of MVMS on the risk of hypertension. MVMS had a lowering effect on systolic blood pressure (SBP) and diastolic blood pressure (DBP): the weighted mean difference (WMD) was −1.31 mmHg (95% CI, −2.48 to −0.14 mmHg) and −0.71 mmHg (95% CI, −1.43 to 0.00 mmHg), respectively. Subgroup analysis indicated that the lowering effect of MVMS on blood pressure was only significant in 134 subjects with chronic disease but not in 1580 healthy subjects, and the WMD for systolic blood pressure (SBP) and DBP in subjects with chronic disease was −6.29 mmHg (95% CI, −11.09 to −1.50 mmHg) and −2.32 mmHg (95% CI, −4.50 to −0.13 mmHg), respectively. The effect size of MVMS on SBP in 58 hypertensive subjects (WMD, −7.98 mmHg; 95% CI, −14.95 to −1.02 mmHg) was more than six times of that in 1656 normotensive subjects (WMD, −1.25 mmHg; 95% CI, −2.48 to −0.02 mmHg). However, no significant effect on DBP was observed in both hypertensive and normotensive subgroups. There was no significant effect of MVMS on risk of hypertension in 22,852 subjects with a normal blood pressure at baseline. In conclusion, although MVMS had a significant lowering effect on blood pressure in normotensive subjects, the lowering effect was too small to effectively prevent future hypertension. MVMS may be an effective method for blood pressure control in subjects with chronic disease including hypertension, but the sample size of subjects with hypertension or other chronic disease was too small, and more well-designed RCTs are needed to confirm this result.

## 1. Introduction

It has been estimated that about 1 billion people suffer from hypertension all over the world [1]. Increased blood pressure is the most important risk factor for premature death, stroke and heart disease [2]. Since pharmaceutical therapy always has a series of adverse effects [3,4], nutrition therapy has received more and more attention in recent years. Previous animal studies have indicated that vitamins and minerals can directly influence blood pressure by modulating sympathetic activity [5], vascular endothelial and smooth muscle function [6], vascular membrane ion exchange [7], and vascular tone [8]. Several randomized controlled trials (RCTs) have been conducted to evaluate the effect of multivitamin and multimineral supplementation (MVMS) on blood pressure and risk of hypertension, but the results were not consistent [9,10,11,12,13,14,15,16,17,18,19,20]. One previous study found that MVMS significantly lowered systolic blood pressure (SBP) and diastolic blood pressure (DBP) in hypertensive subjects but not in normotensive subjects [15]. The lowering effect of MVMS on blood pressure in hypertensive subjects was also observed in another study [13]. Several other studies on subjects with chronic or infectious disease, such as obesity [11], esophageal dysplasia [9] and HIV-infection [19], also observed a significant lowering effect of MVMS on blood pressure or risk of hypertension. However, almost all studies in healthy subjects found no significant effect of MVMS on blood pressure or risk of hypertension [10,12,16,20] except one study which found SBP was significantly lowered by MVMS [17]. Therefore, the difference in the healthy status of included subjects between studies may be one reason for the inconsistent result concerning the effect of MVMS on blood pressure. One study reported that MVMS significantly lowered the risk of hypertension only in males but not females, indicating that a gender difference may exist in the effect of MVMS on blood pressure [9]. Two studies used only multimineral as intervention [13,16], two studies used only multivitamin as intervention [17,19], and the remaining eight studies used the combination of multivitamin and multimineral as intervention [9,10,11,12,14,15,18,20], which may be another reason for the inconsistent results. In addition, the number of vitamins and minerals used for intervention, the age of subjects and the duration of intervention in these studies ranged from 3 to 29, from 21.9 to 64.7 years and from 1 to 86.4 months, respectively. The wide range of these variables among different studies may also contribute to inconsistent results concerning the effect of MVMS on blood pressure. One previous systematic review and meta-analysis based on three RCTs evaluated the combination effect of potassium-magnesium, calcium-magnesium, and calcium-potassium on blood pressure, and no significant result was observed [21]. Recently, the modified Delphi consensus panel report discussed the effect of MVMS on human health and several meta-analyses were included in this report, such as meta-analyses evaluating the effect of MVMS on chronic medical conditions (based on cohort studies), cardiovascular disease (based on cohort studies and RCTs), cancer (based on RCTs), and diabetes (based on RCTs) [22]. However, no meta-analysis has been conducted to systematically evaluate the effect of MVMS (containing at least three vitamins or minerals [22,23]) on blood pressure or risk of hypertension.

There are also several concerns about the safety of long-term MVMS. The Iowa Women’s Health Study indicated that vitamins and minerals supplementation was significantly associated with increased risk of total mortality [24]. One case study indicated that MVMS may be associated with the development of acute hepatitis in an elderly white woman [25]. A Health and Diet Survey conducted by The US Food and Drug Administration found that subjects supplemented with multivitamins/minerals plus single vitamin/mineral and herb/botanical were more likely to report adverse effects, such as blood pressure problem, abdominal pain, nausea, vomiting, allergy, dizziness, itching and rash [26]. These concerns about the possible adverse effects of MVMS make it more urgent to systematically evaluate the efficiency of MVMS on blood pressure.

The aim of the present study was to systematically evaluate the effect of MVMS on blood pressure and risk of hypertension by a meta-analysis of RCTs.

## 2. Materials and Methods

### 2.1. Data Source and Study Selection

We searched Pubmed, Embase and Cochrane Library for the terms (vitamin OR mineral OR multivitamin OR multimineral OR antioxidant OR anti-oxidant OR micronutrient) in combination with (hypertension OR systolic blood pressure (SBP) OR diastolic blood pressure (DBP) OR mean artery pressure (MAP) OR blood pressure) up to May 2018. Hand-searching of the references of relevant articles and recent reviews was undertaken. To be eligible for inclusion, studies must have a randomized controlled design and report data on SBP, DBP, MAP or risk of hypertension. MVMS was defined as a supplement containing ≥3 vitamins or minerals, except when supplements contained ≥3 B vitamins only, because B vitamins exert similar biological actions [23].

### 2.2. Data Extraction and Quality Assessment

Study selection and data extraction were undertaken independently by two investigators, with discrepancies resolved by consensus. The data collected included the name of the first author and publication year, number of subjects in trial and control groups, mean and standard deviation of SBP, DBP and MAP at baseline and endpoint or their changes from baseline to endpoint, odds ratio (OR) (or relative risk (RR) or hazard ratio (HR)) and corresponding 95% confidence interval (95% CI) of hypertension. Data about age, sex, body mass index (BMI), healthy status and race of subjects, study design, duration, and number of vitamins and minerals used for intervention were also extracted.

Review manager 5.3 was used to assess the quality of studies included in the present meta-analysis based on Cochrane criteria, including sequence generation, allocation concealment, blinding of participants, personnel and outcome assessors, incomplete outcome data, selective outcome reporting and other potential risk of bias within a study [27].

### 2.3. Statistical Analysis

For studies evaluating the effect of MVMS on SBP, DBP and MAP, the changes from baseline to endpoint and their corresponding SDs in trial group and control group were used to calculate the effect size, that is, weighted mean difference (WMD). If SD for change was not reported, it was imputed based on SDs at baseline and endpoint by the method in a previous study [28]. For studies that did not report baseline data of SBP, DBP an MAP, mean and SD of SBP, DBP and MAP in trial and control groups at endpoint of intervention were used for data analysis [29]. For crossover study, mean difference between the levels of SBP, DBP and MAP at the end of two intervention periods was used for data analysis [27]. For studies evaluating the effect of MVMS on risk of hypertension, OR and its corresponding 95% CI was used to calculate the overall effect size; if a study presented results as RR or HR, they were considered as OR as a previous study did [30]; if OR, RR or HR were all not reported, the number of subjects with or without hypertension in trial and control groups were imputed into Stata 11.0 to calculate OR and 95% CI. Heterogeneity was assessed by the chi-square method, and I^2^ > 50% indicated significant heterogeneity [27]. A fixed-effect model was used to pool study-specific effect sizes if the *p* value for heterogeneity was >0.1, while a random-effect model was used if the *p* value for heterogeneity was ≤0.1 [31]. For studies including two or more independent comparisons, they were included in the present meta-analysis as if they were from different studies [27]. Meta-regression analysis and restricted cubic spline analysis (3 knots) were used to evaluate the linear and nonlinear relationship between effect size and continuous covariates, respectively [27]. Subgroup analysis was used to evaluate the influence of categorical confounding factors on effect size. When less than 10 studies (or comparisons) reported data of a continuous covariate, meta-regression analysis and restricted cubic spline analysis were not conducted due to lack of power, and subgroup analysis was used to evaluate its influence on effect size by separating studies into different subgroups with a cut-off point. Sensitivity analysis was conducted to evaluate the changes of effect size before and after excluding studies with a high risk of bias in at least one category. Publication bias was evaluated by funnel plot as well as Begg’s and Egger’s tests. A *p* value < 0.05 was considered statistically significant. All data analyses above were conducted by Stata 11.0.

## 3. Results

### 3.1. Characteristics of Included Studies

**Figure 1:**
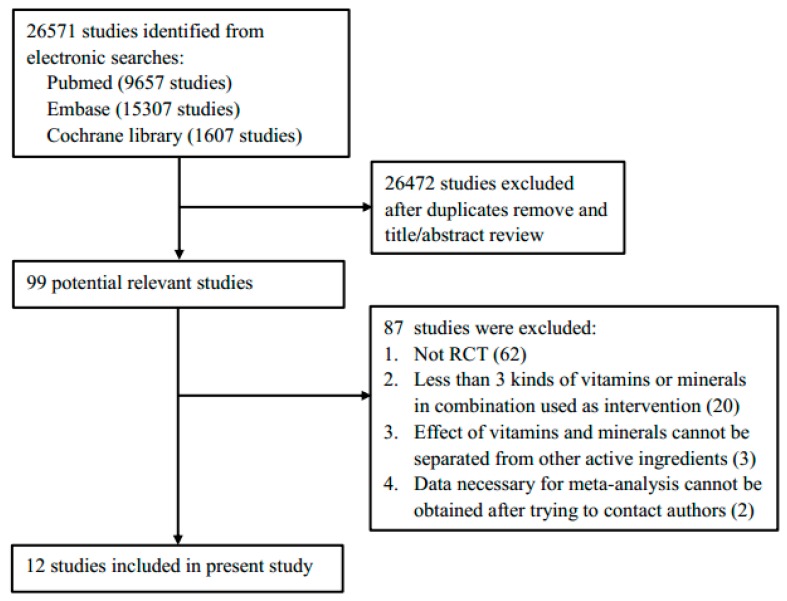
Flow chart for identifying eligible studies.

Figure 1 shows the process for identifying eligible studies. The electronic searches identified 26,571 studies, of which 12 RCTs comprised of 16 independent comparisons and 23,207 subjects were included in the present meta-analysis [9,10,11,12,13,14,15,16,17,18,19,20]. Characteristics of included studies are shown in Table 1 and Table 2. The age of subjects ranged from 21.9 to 64.7 years. Three RCTs including four independent comparisons recruited Chinese as subjects [9,11,14], eight RCTs including 11 independent comparisons recruited Caucasian as subjects [10,12,13,15,16,17,18,20], while one RCT recruited African as subjects [19]. Ten independent comparisons from eight RCTs (2011 subjects) evaluated the effect of MVMS on SBP and DBP [10,11,12,13,15,16,17,18], including two comparisons in hypertensive subjects [13,15] and eight comparisons in normotensive subjects [10,11,12,15,16,17,18]. Only one RCT evaluated the effect of MVMS on MAP [17]. Four RCTs including six independent comparisons (21,196 subjects) evaluated the effect of MVMS on the risk of hypertension in subjects with a normal blood pressure at baseline [9,14,19,20]. The details of supplement types were shown in Tables S1 and S2. Eight RCTs used vitamins plus minerals as supplements [9,10,11,12,14,15,18,20], two RCTs used only vitamins as supplements [17,19], and two RCTs used only minerals as supplements [13,16].

### 3.2. Quality Assessment

The risk of bias of included RCTs was shown in Figure S1. Two RCTs including three independent comparisons had a high risk of bias in blinding of participants and personnel (performance bias) [9,18], and one RCT had a high risk of bias due to unbalanced baseline of SBP between experiment group and control group (other bias) [13]. In many RCTs, it was unclear whether a randomized sequence could be foreseen by participants and trialists (allocation concealment) [9,13,16,17], whether drop-out of subjects was due to acceptable reasons or whether intention-to-treat was used in data analysis (attrition bias) [9,13,19]. All the other included RCTs had a low risk of bias [10,11,12,14,15,20].

### 3.3. Effect of MVMS and SBP

**Figure 2:**
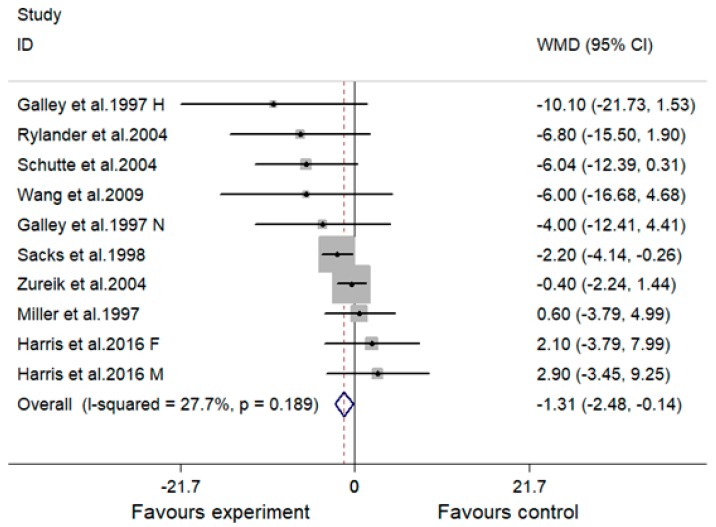
Effect of multivitamin and multimineral supplementation (MVMS) on systolic blood pressure (SBP). WMD, weighted mean difference; CI, confidence interval. Study identities with the same first author’s name and publication year indicate independent comparisons from the same study, and F indicates females, M indicates males, H indicates hypertensive subjects, and N indicates normotensive subjects.

A significant lowering effect of MVMS on SBP was observed: The overall effect size (WMD) was −1.31 mmHg (95% CI, −2.48 to −0.14 mmHg; *p* = 0.028) (Figure 2). No significant heterogeneity was observed between the 10 included comparisons (I^2^ = 27.7%, *p* = 0.189).

**Figure 3:**
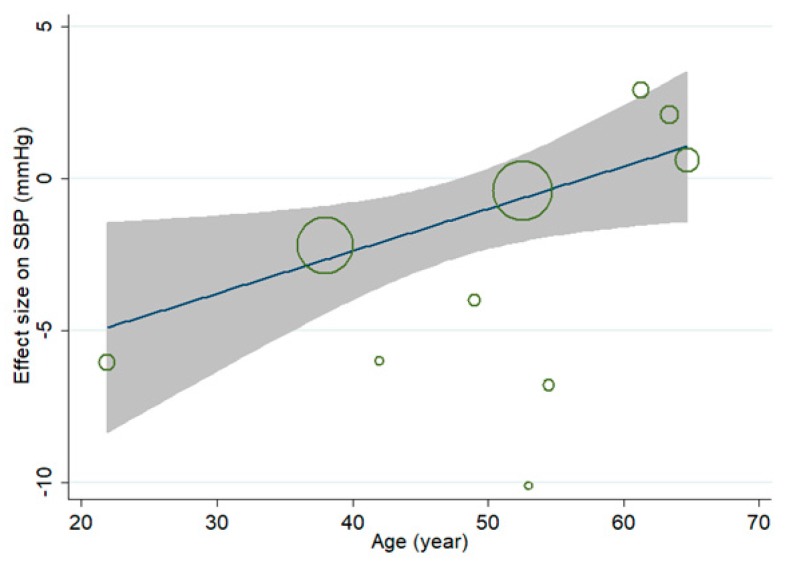
Association between age and effect size of multivitamin and multimineral supplementation (MVMS) on systolic blood pressure (SBP) (weighted mean difference, WMD).

Mete-regression analysis was conducted to evaluate the linear relationship between effect size (WMD) on SBP and continuous covariates. A significant positive association was observed between effect size on SBP and age of subjects (Figure 3), and the coefficient was 0.139 (95% CI, 0.005 to 0.274; *p* = 0.044), indicating that the lowering effect of MVMS on SBP was weaker in older subjects. No significant linear relationship was observed between effect size on SBP and duration, and the coefficient was 0.018 (95% CI, −0.024 to 0.061; *p* = 0.348). Restricted cubic spline analysis indicated that there was no significant nonlinear relationship between effect size on SBP and age (*p* for nonlinearity = 0.201) or duration (*p* for nonlinearity = 0.084). Since less than 10 comparisons reported data of BMI and baseline of SBP, meta-regression and restricted cubic spline analysis were not conducted for them due to lack of power.

Subgroup analysis was conducted to evaluate the association of effect size on SBP with study design (parallel or crossover), sex, BMI (cut-off point = 30 kg/m^2^), race, healthy status (chronic disease or healthy) and baseline blood pressure (hypertension or not) (Table 3). The effect size of MVMS on SBP in hypertensive subjects (WMD, −7.98 mmHg; 95% CI, −14.95 to −1.02 mmHg; *p* = 0.025) was more than six times of that in normotensive subjects (WMD, −1.25 mmHg; 95% CI, −2.48 to −0.02 mmHg; *p* = 0.046). The lowering effect of MVMS on SBP was only significant in subjects with chronic disease (WMD, −6.29 mmHg; 95% CI, −11.08 to −1.50 mmHg; *p* = 0.01) but not in heathy subjects (WMD, −1.13 mmHg; 95% CI, −2.38 to 0.12 mmHg; *p* = 0.077). The lowering effect of MVMS on SBP was significant in Caucasians (WMD, −1.25 mmHg; 95% CI, −2.43 to −0.08 mmHg; *p* = 0.036); only one RCT evaluated the effect of MVMS on SBP in Chinese subjects (obese subjects), and the result was non-significant (WMD, −6.00 mmHg; 95% CI, −16.68 to 4.68 mmHg; *p* = 0.271). No obvious relationship was observed between effect size on SBP and study design, sex or BMI.

### 3.4. Effect of MVMS on DBP

**Figure 4:**
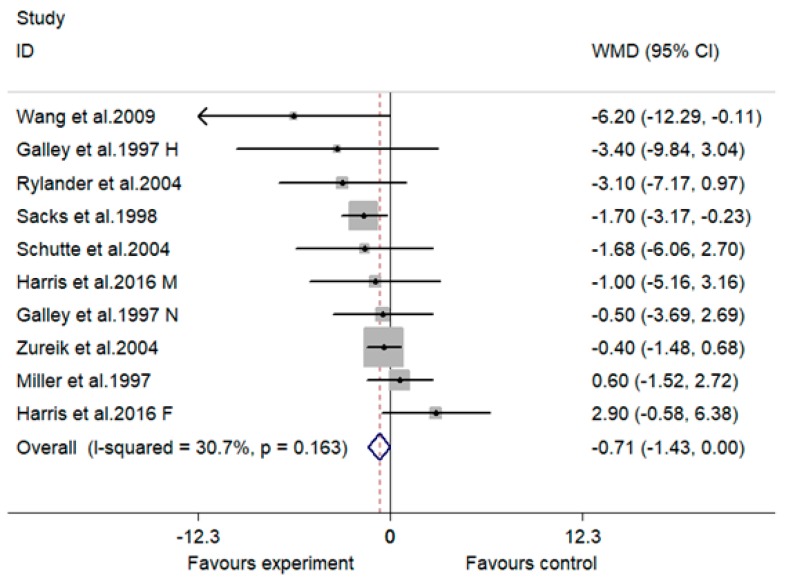
Effect of multivitamin and multimineral supplementation (MVMS) on diastolic blood pressure (DBP). WMD, weighted mean difference; CI, confidence interval. Study identities with the same first author’s name and publication year indicate independent comparisons from the same study; F indicates females, M indicates males, H indicates hypertensive subjects, and N indicates normotensive subjects.

A marginally significant lowering effect of MVMS on DBP was observed: The overall effect size (WMD) was −0.71 mmHg (95% CI, −1.43 to 0.00 mmHg; *p* = 0.051) (Figure 4). No significant heterogeneity was observed between the 10 included comparisons (I^2^ = 30.7%, *p* = 0.163).

**Figure 5:**
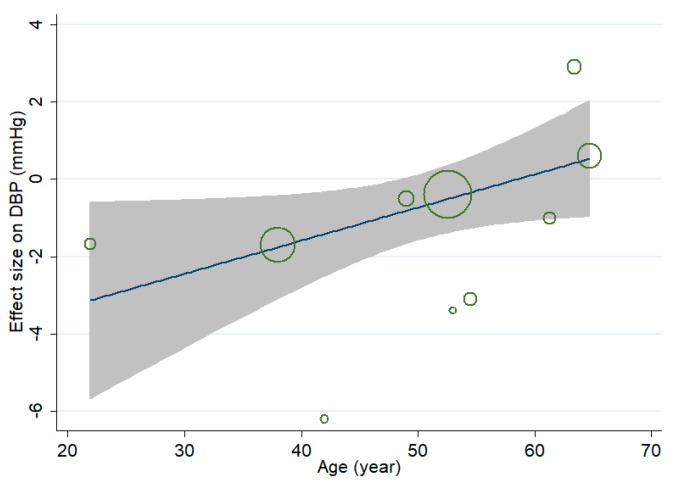
Association between age and effect size of multivitamin and multimineral supplementation (MVMS) on diastolic blood pressure (DBP) (weighted mean difference, WMD).

Mete-regression analysis was conducted to evaluate the linear relationship between effect size (WMD) on DBP and continuous covariates. A marginally significant positive association was observed between effect size on DBP and age of subjects (Figure 5), and the coefficient was 0.086 (95% CI, 0.000 to 0.172; *p* = 0.051), indicating that the lowering effect of MVMS on DBP was weaker in older subjects. No significant linear relationship was observed between effect size on DBP and duration, and the coefficient was 0.006 (95% CI, −0.034 to 0.047; *p* = 0.728). Restricted cubic spline analysis indicated that there was no significant nonlinear relationship between effect size on DBP and age (*p* for nonlinearity = 0.284) or duration (*p* for nonlinearity = 0.580). Since less than 10 comparisons reported data of BMI and baseline, meta-regression and restricted cubic spline analysis were not conducted for them due to lack of power. 

Subgroup analysis was conducted to evaluate the association of effect size on DBP with study design (parallel or crossover), sex, BMI (cut-off point = 30 kg/m^2^), race, healthy status (chronic disease or healthy) and baseline of blood pressure (hypertension or not) (Table 3). The lowering effect of MVMS on DBP was only significant in subjects with chronic disease (WMD, −2.32 mmHg; 95% CI, −4.50 to −0.13 mmHg; *p* = 0.038) but not in heathy subjects (WMD, −0.69 mmHg; 95% CI, −1.50 to 0.13 mmHg; *p* = 0.099). The lowering effect of MVMS on DBP was only significant in obesity subjects (WMD, −6.20 mmHg; 95% CI, −12.29 to −0.11 mmHg; *p* = 0.046) but not in non-obesity subjects (WMD, −0.69 mmHg; 95% CI, −1.50 to 0.13 mmHg; *p* = 0.099). Only one RCT evaluated the effect of MVMS on DBP in Chinese subjects (obese subjects), and a significant lowering effect was observed (WMD, −6.20 mmHg; 95% CI, −12.29 to −0.11 mmHg; *p* = 0.046); no significant effect of MVMS on DBP was observed in Caucasian subjects (WMD, −0.64 mmHg; 95% CI, −1.36 to 0.09 mmHg; *p* = 0.084). No obvious relationship was observed between effect size on DBP and study design, sex or baseline.

### 3.5. Effect of MVMS on MAP

Only one RCT evaluated the effect of MVMS on MAP, and no significant influence was observed (WMD, −3.32 mmHg; 95% CI, −8.41 to 1.77 mmHg; *p* = 0.201).

### 3.6. Effect of MVMS on Risk of Hypertension

**Figure 6:**
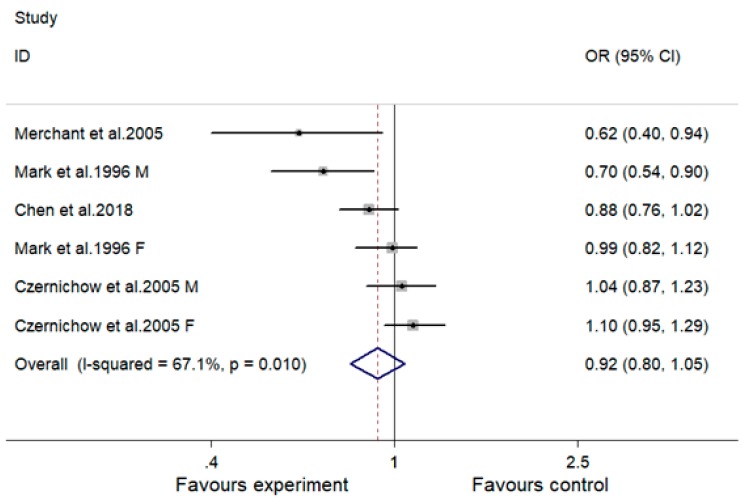
Effect of multivitamin and multimineral supplementation (MVMS) on risk of hypertension. OR, odds ratio; CI, confidence interval. Study identities with the same first author’s name and publication year indicate independent comparisons from the same study; F indicates females, M indicates males.

No significant effect of MVMS on risk of hypertension was observed: The overall effect size (OR) was 0.92 (95% CI, 0.80 to 1.05; *p* = 0.216) (Figure 6). A significant heterogeneity was observed between studies (I^2^ = 67.1%; *p* for heterogeneity = 0.010). Subjects in these studies had normal blood pressure at baseline.

Subgroup analysis indicated that the lowering effect of MVMS on risk of hypertension was significant in one study in African women diagnosed with infectious disease (HIV-positive) (OR, 0.62; 95% 0.40, 0.95; *p* = 0.028) but not in Chinese, Caucasian, healthy subjects or subjects with chronic disease (Table 4). No obvious relationship was observed between effect size on risk of hypertension and duration, age, sex or pregnancy. Since all included studies evaluating the effect of MVMS on risk of hypertension had a parallel design and recruited subjects with normal BMI and normal blood pressure at baseline, subgroup analysis was not conducted on these parameters. Subgroup analysis did not obviously decrease heterogeneity between these studies (Table 4). Meta-regression and restricted cubic spline analyses were also not conducted due to there being less than 10 relevant comparisons included.

### 3.7. Sensitivity Analysis

Sensitivity analysis was conducted to evaluate the effect of studies with a high risk of bias on overall effect size. After excluding studies with a high risk of bias, the lowering effect of MVMS on DBP slightly changed from being marginally significant (WMD, −0.71 mmHg; 95% CI, −1.43 to 0.00 mmHg; *p* = 0.051) to significant (WMD, −0.89 mmHg; 95% CI, −1.65 to −0.12 mmHg; *p* = 0.023), the effect of MVMS on SBP still remained significant (WMD, −1.35 mmHg; 95% CI, −2.57 to −0.13 mmHg; *p* = 0.030), and the effect of MVMS on risk of hypertension still remained non-significant (OR, 0.95; 95% CI, 0.80 to 1.12; *p* = 0.538), indicating that risk of bias did not substantially influence the final results.

### 3.8. Publication Bias

No publication bias was identified among studies evaluating the effect of MVMS on SBP, DBP or risk of hypertension by funnel plots (Figures S2–S4). Consistent results were also observed from Begg’s and Egger’s tests (all *p* values > 0.1). This section may be divided by subheadings. It should provide a concise and precise description of the experimental results, their interpretation, as well as the experimental conclusions that can be drawn.

## 4. Discussion

To our knowledge, this is the first meta-analysis to systematically evaluate the effect of MVMS on blood pressure. The present meta-analysis included 12 randomized controlled trials (RCTs), of which eight RCTs comprised of 10 independent comparisons and 2011 subjects evaluated the effect of MVMS on blood pressure and four RCTs comprised of six independent comparisons and 21,196 subjects evaluated the effect of MVMS on risk of hypertension.

In the present study, we observed a lowering effect of MVMS on SBP and DBP. The lowering effect of MVMS on blood pressure has a biological basis. Previous animal studies indicated that vitamins and minerals can directly influence blood pressure by modulating sympathetic activity [5], vascular endothelial and smooth muscle function [6], vascular membrane ion exchange [7], and vascular tone [8]. In addition, vitamins and minerals may also indirectly influence blood pressure by modulating inflammation and oxidative stress. C-reactive protein (CRP), an acute phase reactant protein, is an independent risk factor for cardiovascular disease including hypertension [32,33], and previous RCTs indicated that vitamins and minerals had a significant lowering effect on CRP [11,34]. Oxidative stress is another important factor contributing to hypertension [35,36], and many vitamins and minerals play an important role in antioxidation, such as vitamin C, vitamin E and selenium. These evidences above can help explain the mechanism by which MVMS lowers blood pressure.

Meta-regression analysis showed a positive linear relationship between the age of subjects and effect size of MVMS on SBP and DBP, indicating that the lowering effect of MVMS on blood pressure became weaker in older subjects. This may be attributed to decreased bioavailability of micronutrients with age. It has been reported that vitamin D biosynthesis in skin, vitamin D receptors in the intestinal epithelial cell and intestinal absorption of vitamin D all decreased in older subjects; conversion of 25-hydroxyvitamin D to the active hormonal form 1,25-dihydroxyvitamin D was also impaired in older subjects [37]. Decreased bioavailability of vitamin D may also limit calcium absorption. The decreased absorption of calcium in the elderly was indeed reported by previous a study [38]. In addition, a previous study indicated that hyperhomocysteinemia and B vitamin deficiency became more prevalent with increased age [39], indicating a decreased bioavailability of B vitamins in older subjects. Decreased bioavailability with age was also observed for other micronutrients, such as magnesium and zinc [40,41]. Therefore, decreased bioavailability of vitamins and minerals may weaken the effect of MVMS on blood pressure in elderly.

Subgroup analysis indicated that the lowering effect of MVMS was only significant in subjects with chronic disease but not in healthy subjects. The effect size on SBP (−6.29 mmHg) and DBP (−2.32 mmHg) in subjects with chronic disease was about five times and three times of that observed in healthy subjects as well as all subjects, respectively. The subgroup of chronic disease included three types of subjects: obese subjects (obese subjects were included into the subgroup of chronic disease since obesity is closely related to a series of chronic diseases including hypertension), hypertensive subjects and subjects with dyspepsia or other gastrointestinal disease. Subgroup analysis according to blood pressure at baseline also found that the lowering effect of MVMS on SBP in hypertensive subjects was more than six times of that in normotensive subjects. Wang et al. evaluated the effect of MVMS on blood pressure in obese subjects, and explained the mechanism for the lowering effect of MVMS on blood pressure in obese subjects as follows: Since many vitamins and minerals play an important role in regulating energy metabolism, higher energy intake in obese subjects may need more vitamins and minerals for energy metabolism compared with normal subjects and thus leads to insufficient vitamins and minerals for blood pressure control [11]. Analogously, many chronic diseases are always accompanied by higher oxidative stress and chronic inflammation; considering that many vitamins and minerals also have antioxidative and anti-inflammatory effects (as discussed in the second paragraph of discussion), higher levesl of oxidative stress and inflammation may in turn expend more vitamins and minerals and thus lead to insufficient vitamins and minerals for blood pressure control. In addition, some medicine taken by subjects with chronic disease, such as proton pump inhibitors for treating gastrointestinal disorders may also lead to vitamin and mineral deficiency [42]. And previous studies have indeed observed that vitamin and mineral deficiency is prevalent in many chronic diseases, such as cardiovascular disease and obesity [42,43,44,45]. Therefore, subjects with chronic disease may need more vitamins and minerals than healthy subjects for blood pressure control, and MVMS for subjects with chronic disease exactly solves this problem. These points may help explain the greater lowering effect of MVMS on blood pressure in subjects with chronic disease than healthy subjects.

In the present study, MVMS had no significant influence on the risk of hypertension. It seems contradictory with the results observed for SBP and DBP. However, this result is easy to understand if we take into consideration that all the studies evaluating the effect of MVMS on risk of hypertension were conducted in subjects with a normal blood pressure at baseline. Subgroup analysis for SBP and DBP indicated that the lowering effect of MVMS on SBP was much weaker in normotensive subjects (only −1.25 mmHg) than in hypertensive subjects (−7.98 mmHg) and that the lowering effect of MVMS on DBP was only −0.77 mmHg (marginally significant) in normotensive subjects. Therefore, the effect size of MVMS on blood pressure in normotensive subjects may be too small to effectively prevent future hypertension. The reason why a non-significant effect of MVMS on DBP was observed in hypertensive subjects was that those hypertensive subjects only had a much higher SBP, but DBP was in the normal range or only slightly higher than 90 mmHg, which was very close to that in normotensive subjects. Subgroup analysis indicated that only one study in HIV-positive African subjects reported a significant lowering effect of MVMS on the risk of hypertension. Previous studies have demonstrated that vitamin and mineral deficiency is very prevalent in HIV-infected patients [46]. Considering the important role of vitamins and minerals in blood pressure regulation, MVMS for these subjects has thus effectively lowered the risk of hypertension. This was, to some extent, consistent with the result that MVMS significantly lowered SBP and DBP by 6.29 and 2.32 mmHg, respectively, in subjects with chronic disease, who were also usually at a high risk of vitamin and mineral deficiency.

The difference of supplement between studies may be another reason for the inconsistent results concerning the effect of MVMS on blood pressure. Two studies used only vitamins as supplement [17,19]. The subjects of the two studies were all normotensive, and both supplements contained antioxidative vitamins (vitamin C and E) and folic acid. Interestingly, both studies reported a significant lowering effect on blood pressure or risk of hypertension, suggesting that vitamin C, vitamin E or folic acid may be effective components in MVMS for blood pressure control. Indeed, three studies without folic acid supplementation reported a non-significant effect on blood pressure in normotensive subjects [10,18,20]. However, the study by Chen et al. indicated that MVMS containing folic acid had no significant influence on risk of hypertension in subjects with a normal blood pressure at baseline compared with the control group [14]. This is easy to understand if we take it into consideration that the control group was also supplemented with folic acid. The reason why no significant effect of MVMS containing folic acid on blood pressure was observed in another study by Harris et al. [12] may be that the normotensive subjects in this study were much older than those in the two studies using only vitamins (containing folic acid) as supplement [17,19]. As we have discussed in the third paragraph of the discussion section, the bioavailability of micronutrients decreased and the lowering effect of MVMS on blood pressure became weaker with aging. In the present meta-analysis, no included studies evaluated the effect of MVMS containing folic acid on blood pressure in hypertensive subjects, but one previous meta-analysis based on RCTs indicated that folic acid in combination with antihypertension drugs has a synergic effect on both SBP and DBP in hypertensive subjects compared with antihypertension drugs alone [47]. The lowering effect of folic acid on blood pressure may be attributed to its important role in homocysteine (Hcy) metabolism. Folic acid can be transformed into 5-methyltetrahydrofolate, a methyl donor for remethylation of Hcy to methione [48]. The lowering effect of folic acid on Hcy has been demonstrated [49]. Higher homocysteine level was associated with higher risk of hypertension, ischaemic heart disease, stroke and other cardiovascular disease [50,51]. One included RCT reported that the lowering effect of antioxidative vitamins and minerals (vitamin E, beta-carotene, vitamin C and zinc) on blood pressure was only significant in hypertensive subjects but not normotensive subjects [15]. This finding was also supported by other included RCTs in the present meta-analysis which consistently reported a non-significant effect of MVMS containing antioxidative vitamins and minerals (but no folic acid) on blood pressure or risk of hypertension in subjects with a normal blood pressure at baseline [10,14,18,20]. Previous studies have indicated that antioxidative vitamins also have a protective effect on other cardiovascular diseases, such as myocardial infarction, peripheral vascular disease and unstable angina [52]. Excessive production of reactive oxygen species (ROS) contributes to hypertension and scavenging of ROS decreases blood pressure [53]; in addition, increased ROS production was observed in hypertension [54]. The association between oxidative stress and other cardiovascular diseases, such as atherosclerosis, heart failure and ischemical reperfusion myocardial injury, have also been demonstrated [55]. This may be the reason why antioxidative vitamins and minerals could lower blood pressure of hypertensive subjects and protect against other cardiovascular diseases. Two included RCTs only used minerals as intervention: One reported that supplementation of calcium + potassium + magnesium had no significant influence on blood pressure compared with placebo [16]; another RCT reported that the supplementation of eight minerals (calcium, potassium, magnesium, sodium, sulphur, chloride, fluoride, silicon) led to a significantly lower blood pressure compared with baseline [13]. A previous review indicated that a series of studies found a significant lowering effect of individual calcium, potassium or magnesium supplement on blood pressure but a non-significant result was also reported [56]. One previous systematic review and meta-analysis based on three RCTs evaluated the combination effect of potassium-magnesium, calcium-magnesium, and calcium-potassium on blood pressure, and no significant result was observed [21]. Since the effect of MVMS on blood pressure in hypertensive subjects was different from normotensive ones (as discussed in the paragraph above), it seems hard to judge whether the combination of more different types of minerals had a better effect on blood pressure control based on the two included RCTs mentioned above [13,16]. When comparing the study by Schutte et al. [17] (supplement: antioxidatvie vitamins + folic acid) with the studies by Wang et al. [11] and Harris et al. [12] (supplement: antioxidatvie vitamins + folic acid + other B vitamins + vitamin A + vitamin D + vitamin K + multimineral), we observed that additional supplementation of minerals and other vitamins on the basis of folic acid and antioxidative vitamins did not show a better lowering effect on blood pressure compared with supplementation of only folic acid and antioxidative vitamins. A similar phenomenon concerning the effect of MVMS on risk of hypertension was also observed when we compared the study by Merchant et al. [19] (supplement: folic acid + other B vitamins + antioxidative vitamins) with the study by Mark et al. [9] (supplement: antioxidatvie vitamins + folic acid + other B vitamins + vitamin A + vitamin D + multimineral). One noteworthy point is that the studies evaluating the effect of MVMS on blood pressure presented results in different ways (difference between baseline and endpoint within group or difference between groups), and all studies reporting a significant lowering effect of MVMS on blood pressure presented the result as a difference between baseline and endpoint within group [11,13,15,17]. When we compared the difference in changes of blood pressure from baseline to endpoint between groups in the present meta-analysis (Figure 2 and Figure 4) (as suggested by the Cochrane Handbook for Systematic review [29]), the results of all four of these studies became non-significant except DBP in the study by Wang et al. [11], and the result of SBP and DBP in the study by Sack et al. became significant [16], indicating that a difference in statistical method may also lead to an inconsistent conclusion.

The present study has several strengths. Firstly, we conducted meta-analysis based on data from RCTs, which provided a high level of evidence for the effect of MVMS on blood pressure. Secondly, eight RCTs comprised of 10 independent comparisons and 2011 subjects evaluated the effect of MVMS on blood pressure, and four RCTs comprised of six independent comparisons and 21,196 subjects evaluated the effect of MVMS on the risk of hypertension. The large sample size increased the statistical power and made our results more plausible. However, several limitations also existed. Firstly, three RCTs with a high risk of within-study bias were included. However, sensitivity analysis indicated that excluding these studies or not did not substantially influence the final results. Secondly, a significant heterogeneity was observed between studies evaluating the effect of MVMS on risk of hypertension, and subgroup analysis did not significantly lower this heterogeneity. However, only one study in HIV-positive Africans reported a significant lowering effect and all the other studies showed a non-significant result. The sample size of this study only accounts for about 4.5% of that in all studies assessing the effect of MVMS on risk of hypertension; a possible reason for this different result has been discussed. Excluding this study or not did not change the final result, indicating that the result was stable and plausible. Thirdly, although a great lowering effect of MVMS on blood pressure was observed in subjects with chronic disease including hypertension, the sample size of subjects with hypertension or other chronic diseases was too small, and thus more well-designed RCTs were needed to confirm whether MVMS would be an effective method for blood pressure control in these subjects. Fourthly, the nature of micronutrient interventions varied greatly across trials with different doses and combinations of vitamins and minerals. Although previous meta-analyses also combined studies with different micronutrients together to obtain an overall effect size of MVMS, it is still unclear whether it is logical to do so because there are a number of different mechanisms through which blood pressure could be affected.

## 5. Conclusions

Although MVMS had a significant lowering effect on blood pressure in normotensive subjects, the lowering effect was too small to effectively prevent future hypertension. MVMS may be an effective method for blood pressure control in subjects with chronic disease including hypertension, but the sample size of subjects with hypertension or other chronic disease was too small, and more well-designed RCTs are needed to confirm this result.

## Figures and Tables

**Table 1:** Characteristics of included RCTs evaluating the effect of MVMS on blood pressure.

Study	Design	Sample Size	Duration (Month)	Age (Year)	Sex	BMI	Race	Healthy Status	Baseline	
									SBP	DBP
Zureik et al., 2004 [10]	P	1162	86.4	52.6	M + F	24.85	Caucasian	Healthy	125.5	81.3
Wang et al., 2009 [11]	P	59	6.5	42	F	30.9	Chinese	Obesity	128	85
Harris et al., 2016 M [12]	P	79	4	61.3	M	27.4	Caucasian	Healthy	137.5	84.1
Harris et al., 2016 F [12]	P	160	4	63.4	F	26.5	Caucasian	Healthy	134.9	82.7
Rylander et al., 2004 [13]	P	37	1	54.5	NR	NR	Caucasian	Hypertension	152.7	91.1
Galley et al., 1997 H [15]	C	21	2	53	NR	NR	Caucasian	Hypertension	165	88.9
Galley et al., 1997 N [15]	C	17	2	49	NR	NR	Caucasian	Dyspepsia or other gastro-intestinal complaints	132.8	79.6
Sacks et al., 1998 [16]	P	148	4	38	NR	23.2	Caucasian	Healthy	115.3	73
Schutte et al., 2004 [17]	P	31	3	21.9	M	25.23	Caucasian	Healthy	128.9	79.1
Miller et al., 1997 [18]	P	297	3	64.7	M + F	NR	Caucasian	NR	NR	NR

NR, not reported. Study identities with the same first author’s name and publication year indicate independent comparisons from the same study, and F indicates females, M indicates males, H indicates hypertensive subjects, and N indicates normotensive subjects; P, parallel design; C, crossover design.

**Table 2:** Characteristics of included RCTs evaluating the effect of MVMS on risk of hypertension.

Study	Design	Sample Size	Duration (Month)	Age (Year)	Sex	BMI	Race	Subjects	Baseline	
									SBP	DBP
Mark et al., 1996 M [9]	P	1461	72	54.5	M	20.39	Chinese	Esophageal dysplasia	131.8	80
Mark et al., 1996 F [9]	P	1857	72	54.5	F	20.39	Chinese	Esophageal dysplasia	131.8	80
Chen et al., 2018 [14]	P	11,837	5	<29 *	F	Normal #	Chinese	Pregnant women	Normal &	Normal &
Merchant et al., 2005 [19]	P	955	5	24.7	F	23.3	African	HIV-positive pregnant women	106	66
Czernichow et al., 2005 M [20]	P	2153	78	52.3	M	25.1	Caucasian	Healthy	Normal &	Normal &
Czernichow et al., 2005 F [20]	P	2933	78	47.8	F	22.8	Caucasian	Healthy	Normal &	Normal &

NR, not reported. Study identities with the same first author’s name and publication year indicate independent comparisons from the same study, and F indicates females, M indicates males; P, parallel design. * Although the mean value of age in this study cannot be obtained, the authors reported that the age of more than 96% subjects was less than 29 years. # Although the mean value of BMI in this study cannot be obtained, the authors reported that the BMI of more than 80% subjects ranged from 18.5 to 24.9 kg/m^2^. & Accurate mean value cannot be obtained.

**Table 3:** Subgroup analysis for the effect of MVMS on SBP and DBP.

Subgroup Analysis	No. of Comparisons	SBP			DBP		
		WMD (95% CI)	I^2^ (%)	*p*	WMD (95% CI)	I^2^ (%)	*p*
Overall	10	−1.31 (−2.48, −0.14)	27.7	0.028	−0.71 (−1.43, 0.00)	30.7	0.051
Design							
Parallel	8	−1.17 (−2.35, 0.02)	28.5	0.053	−0.69 (−1.43, 0.05)	43.1	0.068
Crossover	2	−6.09 (−12.91, 0.72)	0	0.08	−1.07 (−3.93, 1.79)	0	0.463
Sex							
Men	2	−1.57 (−6.06, 2.92)	73.7	0.494	−1.32 (−4.34, 1.70)	0	0.39
Women	2	0.21 (−4.95, 5.37)	41	0.936	0.65 (−2.37, 3.68)	84.5	0.672
Men and Women	2	−0.25 (−1.95, 1.44)	0	0.772	−0.19 (−1.16, 0.77)	0	0.696
Unclear	4	−2.68 (−4.50, −0.86)	0	0.004	−1.71 (−2.96, −0.47)	0	0.007
BMI							
<30 kg/m^2^	5	−1.13 (−2.38, 0.12)	40.9	0.077	−0.69 (−1.50, 0.13)	37.4	0.099
>30 kg/m^2^	1	−6.00 (−16.68, 4.68)	NA	0.271	−6.20 (−12.29, −0.11)	NA	0.046
Unclear	4	−2.20 (−5.59, 1.20)	34.6	0.205	−0.45 (−2.02, 1.12)	11.3	0.571
Race							
Caucasian	9	−1.25 (−2.43, −0.08)	31.6	0.036	−0.64 (−1.36, 0.09)	18.6	0.084
Chinese	1	−6.00 (−16.68, 4.68)	NA	0.271	−6.20 (−12.29, −0.11)	NA	0.046
Healthy status							
Healthy	5	−1.13 (−2.38, 0.12)	40.9	0.077	−0.69 (−1.50, 0.13)	37.4	0.099
Chronic disease	4	−6.29 (−11.09, −1.50)	0	0.01	−2.32 (−4.50, −0.13)	1.9	0.038
Unclear	1	0.60 (−3.79, 4.99)	NA	0.789	0.60 (−1.52, 2.72)	NA	0.579
Baseline of blood pressure							
Normotensive subjects	7	−1.25 (−2.48, −0.02)	24.8	0.046	−0.77 (−1.55, 0.02)	36.9	0.055
Hypertensive subjects	2	−7.98 (−14.95, −1.02)	0	0.025	−3.19 (−6.63, 0.25)	0	0.07
Unclear	1	0.60 (−3.79, 4.99)	NA	0.789	0.60 (−1.52, 2.72)	NA	0.579

NA, no associated with this item. SBP, systolic blood pressure; DBP, diastolic blood pressure; WMD, weighted mean difference; CI, confidence interval.

**Table 4:** Subgroup analysis for the effect of MVMS on risk of hypertension.

Subgroup Analysis	No. of Comparisons	OR (95% CI)	I^2^ (%)	*p*
Overall	6	0.92 (0.80, 1.05)	67.1	0.216
Duration				
<6 months	2	0.78 (0.57, 1.08)	67.4	0.14
≥6 years	4	0.97 (0.83, 1.13)	56.7	0.676
Age				
Young (<30 years)	2	0.78 (0.57, 1.08)	56.7	0.14
Middle aged (40–55 years)	4	0.97 (0.83, 1.13)	67.4	0.676
Sex				
Men	2	0.86 (0.59, 1.27)	84.2	0.457
Women	4	0.94 (0.80, 1.10)	64.9	0.437
Race				
Caucasian	2	1.07 (0.96, 1.20)	0	0.227
Chinese	3	0.87 (0.74, 1.03)	61.6	0.103
African	1	0.62 (0.40, 0.95)	NA	0.028
Healthy status				
Healthy subjects	2	1.07 (0.96, 1.20)	0	0.227
Chronic disease	2	0.85 (0.60, 1.19)	80.6	0.33
Infectious disease	1	0.62 (0.40, 0.95)	NA	0.028
Unclear	1	0.88 (0.76, 1.02)	NA	0.089
Pregnant or not				
Pregnant women	2	0.78 (0.57, 1.08)	56.7	0.14
Normal adults	4	0.97 (0.83, 1.13)	67.4	0.676

NA, no associated with this item. OR, odds ratio; CI, confidence interval.

## Supplementary Materials

The following are available online at http://www.mdpi.com/2072-6643/10/8/1018/s1, Table S1: Details of supplement used in included studies evaluating the effect of MVMS on blood pressure, Table S2: Details of supplement used in included studies evaluating the effect of MVMS on risk of hypertension, Figure S1: Risk of bias of included RCTs, Figure S2: Funnel plot for studies evaluating the effect of MVMS on SBP, Figure S3: Funnel plot for studies evaluating the effect of MVMS on DBP, Figure S4: Funnel plot for studies evaluating the effect of MVMS on risk of hypertension.
