# Supplementary material for: Effects of Multivitamin and Multimineral Supplementation on Blood Pressure: A Meta-Analysis of 12 Randomized Controlled Trials
Source: Nutrients. 2018 Aug 3;10(8):1018. doi: 10.3390/nu10081018 (PMC6116168; doi:10.3390/nu10081018)
Supplement: Supplementary file 1 [file nutrients-10-01018-s001.pdf]

Table S1. Details of supplement used in included studies evaluating the effect of MVMS on blood pressure

Study	Types of supplement	No. of vitamins and minerals	Details for supplement	Control
Zureik et al.2004[10]	Vitamins plus minerals	5(3 vitamins + 2 minerals)	Vitamin C, vitamin E, beta-carotene, selenium, zinc	Placebo
Wang et al.2009[11]	Vitamins plus minerals	29(13 vitamins + 16 minerals)	Vitamin A, vitamin D, vitamin E, vitamin B1, vitamin B2, vitamin B6, vitamin C, vitamin B12, vitamin K1, biotin, folic acid, niacin, pantothenic acid, calcium, phosphorus, chlorine, magnesium, iron, copper, zinc, manganese, iodine, chromium, molybdenum, selenium, nickel, stannum, silicon, vanadium	Placebo
Harris et al.2016 M[12]	Vitamins plus minerals	24(13 vitamins + 11 minerals)	Vitamin A, vitamin E, vitamin B1, vitamin B2, niacin, pantothenic acid, vitamin B6, vitamin B12, vitamin D, biotin, folic acid, vitamin C, vitamin K1, calcium, magnesium, selenium, molybdenum, chromium, manganese, iron, copper, iodine, potassium, zinc	Placebo
Harris et al.2016 F[12]	Vitamins plus minerals	24(13 vitamins + 11 minerals)	Vitamin A, vitamin E, vitamin B1, vitamin B2, niacin, pantothenic acid, vitamin B6, vitamin B12, vitamin D, biotin, folic acid, vitamin C, vitamin K1, calcium, magnesium, selenium, molybdenum, chromium, manganese, iron, copper, iodine, potassium, zinc	Placebo
Rylander et al.2004[13]	Multimineral	8 minerals	Water rich in calcium, magnesium, sodium, potassium, sulphur, chloride, fluoride, silicon	Distilled water
Galley et al.1997 H[15]	Vitamins plus minerals	4(3 vitamins + 1 mineral)	Vitamin E, beta-carotene, vitamin C, zinc	Placebo
Galley et al.1997 N[15]	Vitamins plus minerals	4(3 vitamins + 1 mineral)	Vitamin E, beta-carotene, vitamin C, zinc	Placebo
Sacks et al.1998[16]	Multimineral	3 minerals	Potassium, magnesium, calcium	Placebo
Schutte et al.2004[17]	Multivitamin	3 vitamins	Vitamin C, vitamin E, folic acid	Placebo
Miller et al.1997[18]	Vitamins plus minerals	7(3 vitamins + 4 minerals)	Vitamin E, vitamin C, beta-carotene, zinc, selenium, copper, manganese	Placebo

Study identities with the same first author's name and publication year indicate independent comparisons from the same study, and F indicates females, M indicates males, H indicates hypertensive subjects, and N indicates normotensive subjects.

Table S2. Details of supplement used in included studies evaluating the effect of MVMS on risk of hypertension

Study	Types of supplement	No. of vitamins and minerals	Details for supplement	Control
Mark et al.1996 M[9]	Vitamins plus minerals	26(13 vitamins + 13 minerals)	Beta-carotene, vitamin A, vitamin E, vitamin C, folic acid, vitamin B1, vitmain B2, niacin, vitamin B6, vitamin B12, vitamin D, biotin, pantothenic acid, calcium, phosphorus, iodine, iron, magesium, copper, manganese, potassium, choloride, chromium, molybdenum, selenium, zinc	Placebo
Mark et al.1996 F[9]	Vitamins plus minerals	26(13 vitamins + 13 minerals)	Beta-carotene, vitamin A, vitamin E, vitamin C, folic acid, vitamin B1, vitmain B2, niacin, vitamin B6, vitamin B12, vitamin D, biotin, pantothenic acid, calcium, phosphorus, iodine, iron, magesium, copper, manganese, potassium, choloride, chromium, molybdenum, selenium, zinc	Placebo
Chen et al.2018[14]	Vitamins plus minerals	15(10 vitamins + 5 minerals)	Folic acid, vitamin A, vitamin E, vitamin D, vitamin C, vitamin B1, vitamin B2, vitamin B6, vitamin B12, niacin, zinc, copper, iodine, selenium, iron	Folic acid
Merchant et al.2005[19]	Multivitamin	7 vitamins	Vitamin B1, vitamin B2, vitamin B6, vitamin B12, vitamin C, vitamin E, folic acid	Placebo
Czernichow et al.2005 M[20]	Vitamins plus minerals	5 (3 vitamins and 2 minerals)	vitamin C, vitamin E, beta-carotene, selenium, zinc	Placebo
Czernichow et al.2005 F[20]	Vitamins plus minerals	5 (3 vitamins and 2 minerals)	vitamin C, vitamin E, beta-carotene, selenium, zinc	Placebo

Study identities with the same first author's name and publication year indicate independent comparisons from the same study, and F indicates females, M indicates males.

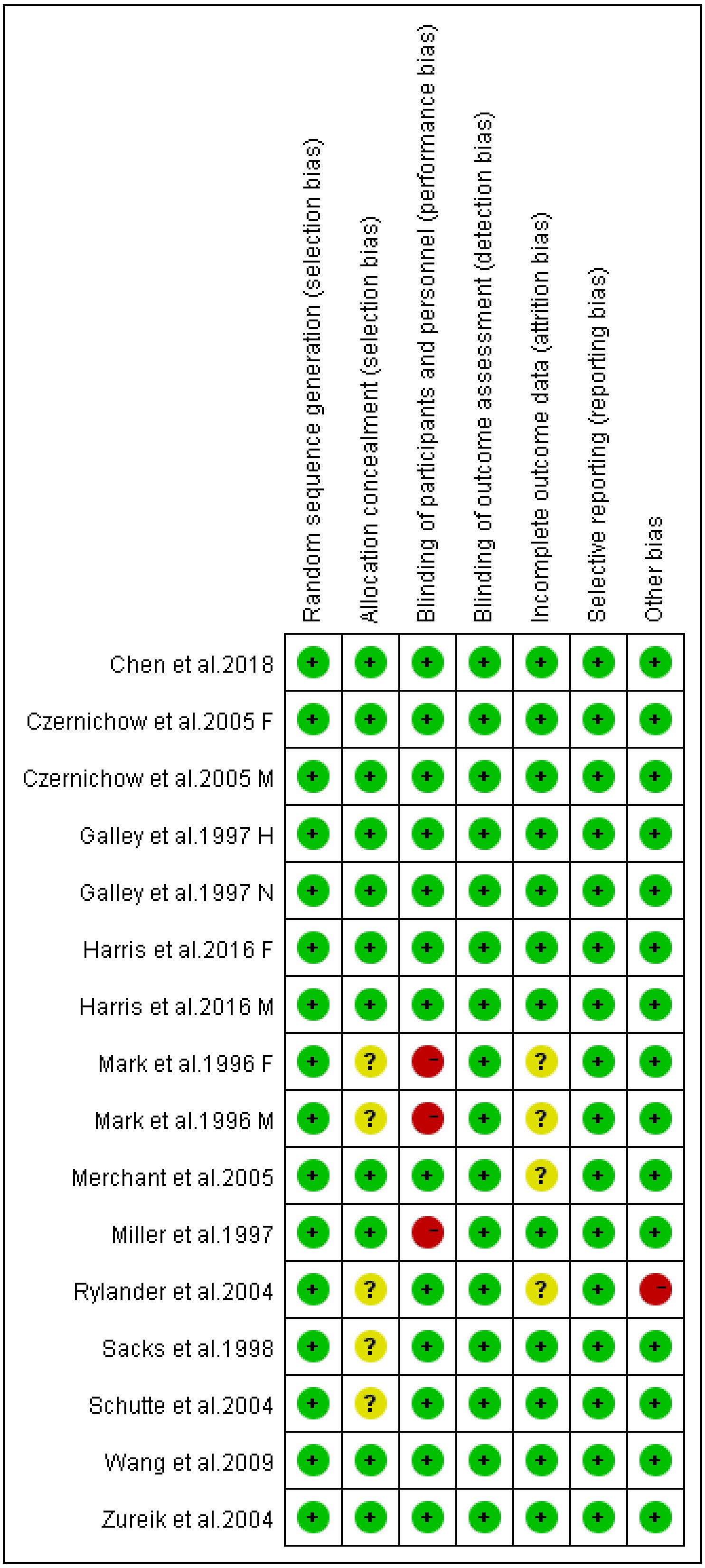

Figure S1. Risk of bias of included RCTs. Red, green and yellow balls indicate high risk of bias, low risk of bias and unclear risk of bias, respectively. Study identities with the same first author's name and publication year indicate independent comparisons from the same study, and F indicates females, M indicates males, H indicates hypertensive subjects, and N indicates normotensive subjects.

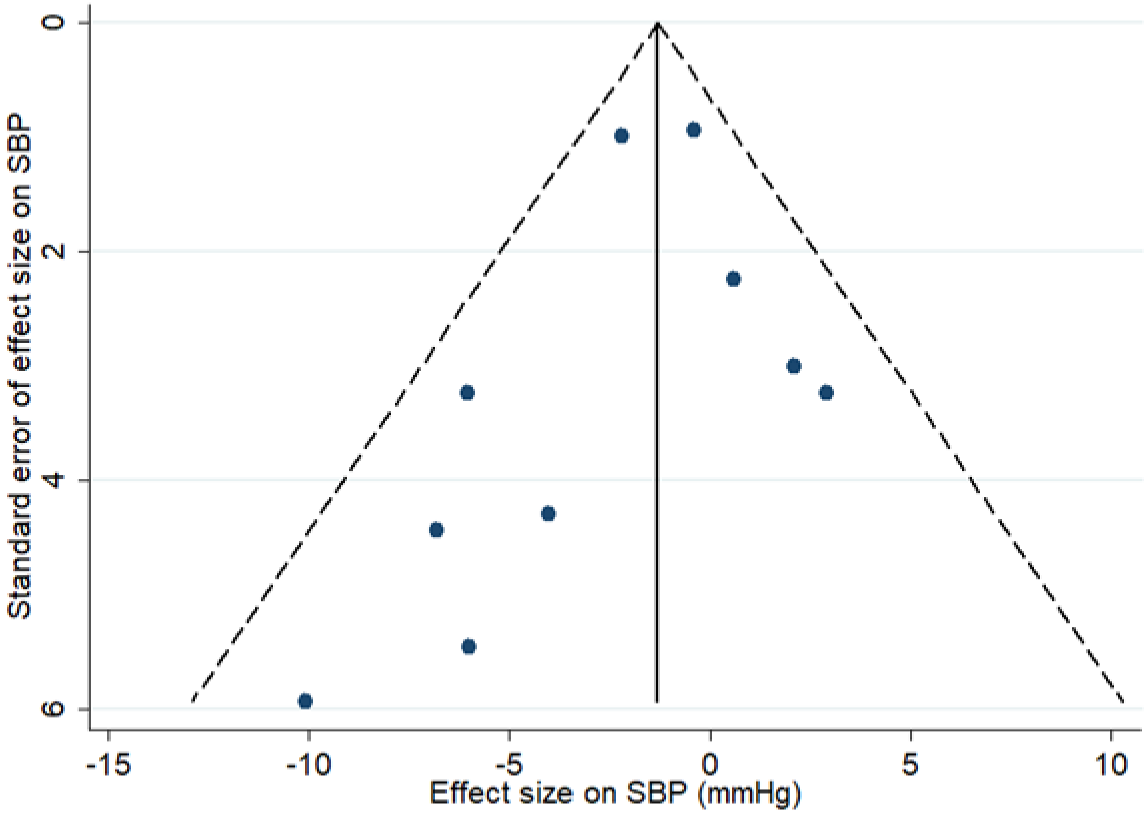

Figue S2. Funnel plot for studies evaluating the effect of multivitamin and multimineral supplementation (MVMS) on systolic blood pressure (SBP).

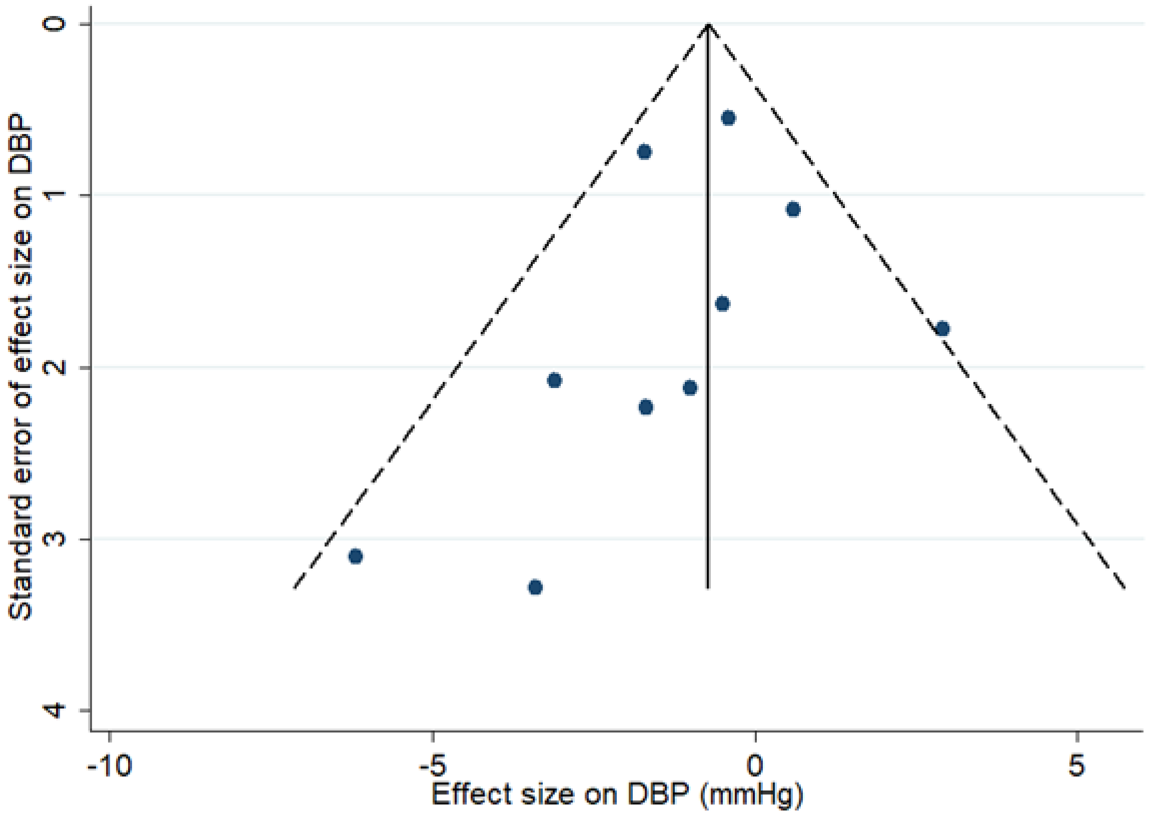

Figue S3. Funnel plot for studies evaluating the effect of multivitamin and multimineral supplementation (MVMS) on diastolic blood pressure (DBP).

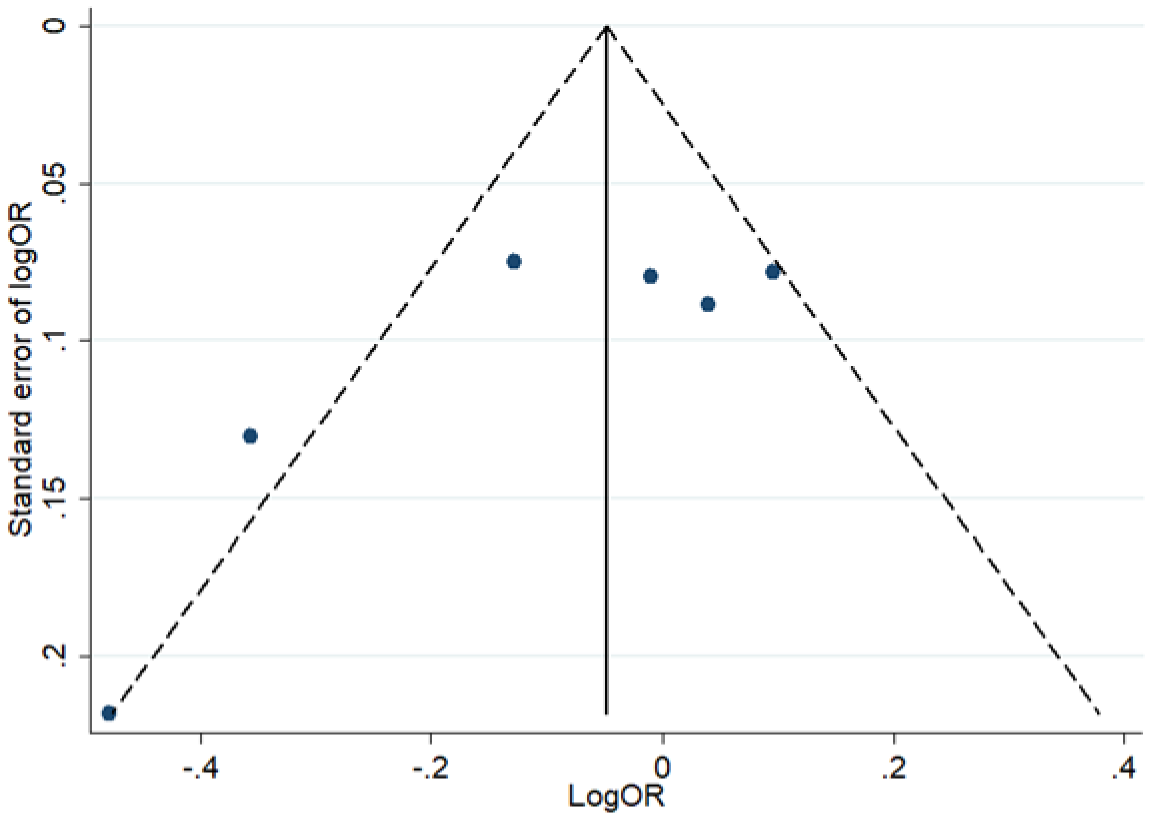

Figue S4. Funnel plot for studies evaluating the effect of multivitamin and multimineral supplementation (MVMS) on risk of hypertension. OR, odds ratio.
